# Supplementary material for: The effect of ascertainment on penetrance estimates for rare variants: Implications for establishing pathogenicity and for genetic counselling
Source: PLoS One. 2023 Sep 21;18(9):e0290336. doi: 10.1371/journal.pone.0290336 (PMC10513297; doi:10.1371/journal.pone.0290336)
Supplement: Attachment — Submitted filename: PLOS_one_response_to_reviews_ascertainment.docx [file pone.0290336.s001.docx]

21 July 2023

Dear Dr Anshuman Mishra, PhD
Academic Editor
PLOS ONE,

Re: PONE-D-23-06786

The effect of ascertainment on penetrance estimates for rare variants: implications for establishing pathogenicity and for genetic counselling

Reponses to reviews:

We thank both reviewers for their recognition of the importance of this work, and the suggestions to improve the layout.

The previous submission was not specifically formatted for PLOS One, so we took advantage of the reviewer’s suggestions to better define the analysis and re-arrange the results and discussion by revising the structure of the manuscript. This has entailed including the previous supplementary text in the main manuscript, as well as major re-organization of the manuscript to make the work easier to understand.

There is no substantively new material in the methods and results, only reorganization and clarification. This has allowed us to address reviewer 1’s points 1 and 2 in the process of rearranging into standard PLOS format.

1. Representation of statistical analysis should be more defined to understand better way.

- See response above.

2. Results should be compared with recent published work and emphasize on betterment cause.

We have added new references to the frequency of coding variants in the genome (ref #21, Gudmundsson et al., 2022), and challenges with interpretation of rare variants in small pedigrees in cardiomyopathy (ref 21; McGurk et al., medrxiv).

3. Result and discussion section should be arranged more properly with the appropriate content.

- See response above

4. Application of the app-research defined in infectious diseases model with healthcare management.

> Infectious disease and healthcare management are beyond the scope of the current manuscript and have not been explicitly addressed.

Please let me know if you require further details,

Best regards,

Andrew Paterson
